# Supplementary material for: Dark wing pigmentation as a mechanism for improved flight efficiency in the Larinae
Source: Commun Biol. 2022 Nov 22;5:1205. doi: 10.1038/s42003-022-04144-8 (PMC9681726; doi:10.1038/s42003-022-04144-8)
Supplement: Supplementary file 1 — Reporting Summary [file 42003_2022_4144_MOESM1_ESM.pdf]

Corresponding author(s): Madeleine Goumas

Last updated by author(s): Oct 7, 2022

Reporting Summary

Nature Portfolio wishes to improve the reproducibility of the work that we publish. This form provides structure for consistency and transparency in reporting. For further information on Nature Portfolio policies, see our Editorial Policies and the Editorial Policy Checklist.

Statistics

For all statistical analyses, confirm that the following items are present in the figure legend, table legend, main text, or Methods section.

n/a	Confirmed	
☐	☒	The exact sample size (*n*) for each experimental group/condition, given as a discrete number and unit of measurement
☐	☒	A statement on whether measurements were taken from distinct samples or whether the same sample was measured repeatedly
☐	☒	The statistical test(s) used AND whether they are one- or two-sided<br>*Only common tests should be described solely by name; describe more complex techniques in the Methods section.*
☐	☒	A description of all covariates tested
☐	☒	A description of any assumptions or corrections, such as tests of normality and adjustment for multiple comparisons
☐	☒	A full description of the statistical parameters including central tendency (e.g. means) or other basic estimates (e.g. regression coefficient) AND variation (e.g. standard deviation) or associated estimates of uncertainty (e.g. confidence intervals)
☐	☒	For null hypothesis testing, the test statistic (e.g. *F*, *t*, *r*) with confidence intervals, effect sizes, degrees of freedom and *P* value noted<br>*Give P values as exact values whenever suitable.*
☒	☐	For Bayesian analysis, information on the choice of priors and Markov chain Monte Carlo settings
☒	☐	For hierarchical and complex designs, identification of the appropriate level for tests and full reporting of outcomes
☐	☒	Estimates of effect sizes (e.g. Cohen's *d*, Pearson's *r*), indicating how they were calculated

Our web collection on statistics for biologists contains articles on many of the points above.

Software and code

Policy information about availability of computer code

Data collection	Two existing datasets were used and cited - supplementary material from Dufour et al. (2020) and AVONET (Tobias et al. 2022). Also BirdTree (Jetz et al. 2012) and Olsen (2018).
Data analysis	R version 4.1.2 with 'phylolm' (Ho & Ane 2014) for the main analysis and 'phytools' (Revell 2012) for visualisation (otherwise base R). The 'glmmTMBphylo' function from the 'glmmTMB' extension (Li & Bolker 2019) was used to run a phylogenetic beta regression.

For manuscripts utilizing custom algorithms or software that are central to the research but not yet described in published literature, software must be made available to editors and reviewers. We strongly encourage code deposition in a community repository (e.g. GitHub). See the Nature Portfolio guidelines for submitting code & software for further information.

Data

Policy information about availability of data

All manuscripts must include a data availability statement. This statement should provide the following information, where applicable:

- Accession codes, unique identifiers, or web links for publicly available datasets
- A description of any restrictions on data availability
- For clinical datasets or third party data, please ensure that the statement adheres to our policy

The data used in this analysis can be found at https://doi.org/10.5281/zenodo.7156454.

Human research participants

Policy information about studies involving human research participants and Sex and Gender in Research.

Reporting on sex and gender	NA
Population characteristics	NA
Recruitment	NA
Ethics oversight	NA

Note that full information on the approval of the study protocol must also be provided in the manuscript.

Field-specific reporting

Please select the one below that is the best fit for your research. If you are not sure, read the appropriate sections before making your selection.

☐ Life sciences ☐ Behavioural & social sciences ☒ Ecological, evolutionary & environmental sciences

For a reference copy of the document with all sections, see nature.com/documents/nr-reporting-summary-flat.pdf

Ecological, evolutionary & environmental sciences study design

All studies must disclose on these points even when the disclosure is negative.

Study description	A phylogenetic comparative analysis of wing darkness in the gull subfamily. The relationship between mantle darkness and species' wing loading and average distance from the equator is tested in a linear model with correction for an Ornstein-Uhlenbeck process of evolution. A phylogenetic beta regression is used to test for a relationship between these explanatory variables and the proportion of black on the wingtips. Finally, a linear model is used to test for a relationship between wing loading and aspect ratio. Tests are two-sided and alpha is set at 0.05.
Research sample	I included all Larinae (gull) species listed by BirdTree (from where the phylogenetic tree is obtained) and which have wing measurements available. This is almost all recognised species of gull (50 species). I concentrated on the Larinae because they are relatively similar in morphology, colouration and ecology.
Sampling strategy	All possible species were included.
Data collection	This was a desk-based study and no data were generated by the author other than the calculations from existing data, which are indicated in the manuscript. The data were obtained from Olsen (2018), Dufour et al. (2020), BirdTree (2012) and AVONET (2022).
Timing and spatial scale	NA - Data were not collected by the author.
Data exclusions	One gull species listed by BirdTree was excluded due to there being no wingspan data from which to calculate wing loading.
Reproducibility	The data and code have been made fully available.
Randomization	NA
Blinding	NA

Did the study involve field work? ☐ Yes ☒ No

Reporting for specific materials, systems and methods

We require information from authors about some types of materials, experimental systems and methods used in many studies. Here, indicate whether each material, system or method listed is relevant to your study. If you are not sure if a list item applies to your research, read the appropriate section before selecting a response.

Materials & experimental systems

n/a	Involved in the study	
☒	☐	Antibodies
☒	☐	Eukaryotic cell lines
☒	☐	Palaeontology and archaeology
☒	☐	Animals and other organisms
☒	☐	Clinical data
☒	☐	Dual use research of concern

Methods

n/a	Involved in the study	
☒	☐	ChIP-seq
☒	☐	Flow cytometry
☒	☐	MRI-based neuroimaging
